# Concurrent Adrenal Neuroblastoma and Kawasaki Disease: A Report of a Rare Case

**DOI:** 10.1155/2013/931703

**Published:** 2013-02-06

**Authors:** Samin Alavi, Alireza Fahimzad, Farzaneh Jadali, Farid Ghazizadeh, Armin Rashidi

**Affiliations:** ^1^Pediatric Congenital Hematologic Disorders Research Center, Mofid Children's Hospital, Shahid Beheshti University of Medical Sciences, Tehran, Iran; ^2^Department of Pediatric Infectious Diseases, Mofid Children's Hospital, Shahid Beheshti University of Medical Sciences, Tehran, Iran; ^3^Department of Pediatric Pathology, Mofid Children's Hospital, Shahid Beheshti University of Medical Sciences, Tehran, Iran; ^4^Department of Internal Medicine, Eastern Virginia Medical School, Norfolk, VA, USA

## Abstract

Kawasaki disease (KD) is a systemic vasculitis of unknown etiology and a leading cause of acquired heart disease. It is assumed that there is an activation of the immune system by an infectious trigger in a genetically susceptible host. Neuroblastoma is the most common extracranial solid tumor in young children. It mainly originates from primordial neural crest cells that generate the adrenal medulla and sympathetic ganglia. A diagnosis of concurrent KD and neuroblastoma in a living child has been made in only one previous report. We report the second case and review the literature.

## 1. Introduction

Kawasaki disease (KD) is an acute vasculitis of childhood that predominantly affects the coronary arteries. The etiology of KD remains unknown; however, an infectious agent is strongly suspected based on clinical and epidemiologic features [1]. It typically affects children younger than 5 years of age. The peak age of incidence of KD is from 6 months to 2 years of age and it is rare in infants ≤3 months old [2]. The highest annual incidence rates of KD are reported from Japan with 206.2 and 239.6 per 100,000 children aged 0 to 4 years in 2009 and 2010, respectively [3].

Neuroblastoma is the most common extracranial solid tumor in children, accounting for 8% to 10% of all childhood cancers. The prevalence is about 1 case per 7,000 live births, and there are about 800 new cases of neuroblastoma per year in the United States [4, 5]. It is the most common cancer diagnosed during infancy and occurs most frequently in children less than 5 years of age, with a median age of 17 months at presentation [6]. We here report an extremely rare case of an infant diagnosed with concurrent Kawasaki disease and neuroblastoma.

## 2. Case Report

**Figure 1:**
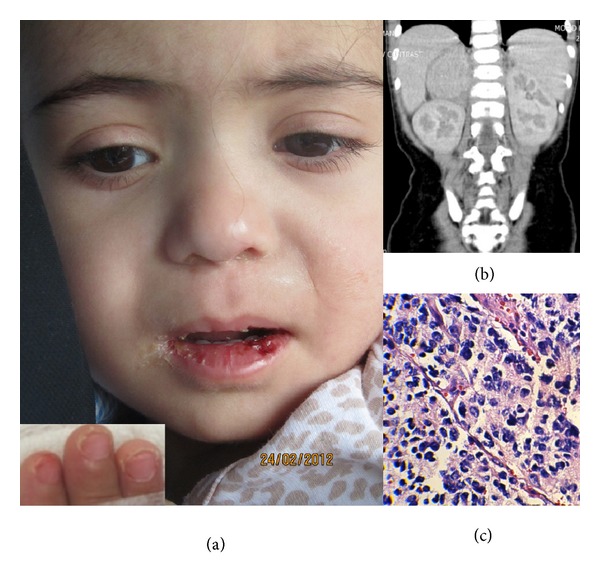
Clinical, radiographic, and pathologic features of a patient with concurrent Kawasaki disease and neuroblastoma. Red fissured lips (a) and desquamation of finger tips (inset). Computed tomography scan showing a mass originating from the right adrenal gland (b). Histopathology showing a tumor composed of predominantly atypical small round cells arranged in sheets, with pleomorphic nuclei, pink cytoplasm, and high mitotic indices (c).

An 18-month-old girl was admitted to our infectious diseases department with a fever for 6 days and puffy red eyes. On physical examination, she was acutely ill, febrile, and irritable. Bulbar conjunctivitis, facial erythema at midline, red fissured lips, and a strawberry tongue were other significant findings (Figure 1(a)). There were also several small bilateral cervical lymphadenopathies, the greatest of which was 1.5 × 1.0 cm. On hospital day 2, mild swelling and desquamation developed on her hands and feet (Figure 1, inset). Transthoracic echocardiography demonstrated a 3 mm dilatation in her right coronary artery, and a diagnosis of Kawasaki disease was made.

She received 2 gr/kg intravenous immunoglobulin (IVIG) for two days as well as aspirin 80 mg/kg/day. Due to the persistence of fever, a computed tomography scan of the brain, chest, abdomen, and pelvis was performed, which was remarkable for a 3 cm × 3 cm abdominal mass originating from the right adrenal gland and with foci of calcification (Figure 1(b)). A 24-hour urine collection showed elevated vanillylmandelic acid levels at 8.5 mg/24 h (normal range: 2–5 mg/24 h). Exploratory laparatomy and total adrenalectomy without any gross residual tumor were performed. The pathology was reported as a stroma-poor undifferentiated neuroblastoma with unfavorable prognosis (Figure 1(c)). MYCN amplification by fluorescence *in situ* hybridization revealed 8 copy numbers of this oncogene. A bone scan as well as a bone marrow aspiration and biopsy were unremarkable. Three days after laparotomy, she developed periungual desquamation over the distal parts of her extremities. Chemotherapy with the N_6_ protocol (Cyclophosphamide/Adriamycin/Vincristine alternating with Cisplatin and VP-16) was initiated. The patient had an unremarkable course and repeat imaging three months later showing no evidence of residual tumor. The patient is free of tumor after 18 months at the time of the report of this case.

## 3. Discussion

Yanagisawa et al. reported a 6-month-old male infant who died of KD and had a small encapsulated mass in the left paravertebral region found on necropsy. The histopathological diagnosis was neuroblastoma [7]. In another study on autopsy results of 61 cases of KD who died between 2 months and 8 years after complete recovery of KD, one patient had a neuroblastoma on autopsy [8]. The case reported here is the second living child reported in the literature with concurrent KD and neuroblastoma. The only other available report is a 4-month-old boy with KD who developed acute urinary retention on the third day of admission and was later found to have a stroma-poor undifferentiated neuroblastoma [9]. Neuroblastoma is one of the malignancies in infants that may be diagnosed as an incidental finding by imaging studies [10, 11], like in our case. Adrenal incidentalomas are slightly more frequent in the right adrenal gland and in females [11]. Our patient was also a girl with a tumor originating from the right adrenal gland.

There are a few reports of cases with malignancy diagnosed following a diagnosis of KD. The malignancies reported are acute lymphoblastic leukemia in 3 cases, Hodgkin's disease in one case, osteosarcoma in one case, Schwannoma in one case, giant cell tumor of the tendon sheath in one case, and malignant reticuloma in one case [12, 13]. Alterations in the immune system have been postulated to underlie KD [14–16]. Whether such alterations predispose to certain types of malignancy as well is unclear. Larger epidemiological studies are required to determine whether the observed associations are mere coincidence or reflect true associations.
